# Evaluation of THP-1 and Jurkat Cell Lines Coculture for the In Vitro Assessment of the Effects of Immunosuppressive Substances

**DOI:** 10.3390/toxics12080607

**Published:** 2024-08-19

**Authors:** Nina Franko, Marija Sollner Dolenc

**Affiliations:** Department of Pharmaceutical Chemistry, Faculty of Pharmacy, University of Ljubljana, SI-1000 Ljubljana, Slovenia; nina.franko@ffa.uni-lj.si

**Keywords:** immunosuppression, coculture, in vitro, new approach method, cell lines, bisphenols

## Abstract

The strong appeal to reduce animal testing calls for the development and validation of in vitro, in chemico and in silico models that would replace the need for in vivo testing and ex vivo materials. A category that requires such new approach methods is the assessment of immunosuppression that can be induced by chemicals including environmental pollutants. To assess the immunosuppressive action on monocytes and lymphocytes, we mimicked the whole-blood cytokine-release assay by preparing an in vitro coculture of THP-1 and Jurkat cell lines. We optimised its activation and investigated the effects of known immunosuppressive drugs with different mechanisms of action on the release of proinflammatory cytokines. Decreased secretion of IL-8 was achieved by several immunosuppressive mechanisms and was therefore selected as an appropriate marker of immunosuppression. A set of environmentally occurring bisphenols, BPA, BPAP, BPP, BPZ, BPE, TCBPA and BPS-MAE, were then applied to the model and BPP and BPZ were found to act as potent immunosuppressants at micromolar concentrations.

## 1. Introduction

The immune system is a complex and highly specialised network of organs, cells and functional proteins that protect the body from pathogenic bacteria, viruses and malignant cells. The elimination of pathogenic, toxic and allergenic particles by the immune system is enabled by its ability to differentiate between endogenous and exogenous antigens [1]. Any failure to recognise (non-)self-antigens, as well as unbalanced activation or regulation of immune pathways during pathogen surveillance or clearance, results in an inappropriate immune response [2]. When self-tolerance fails, inappropriate immune stimulation may lead to autoimmune diseases, while increased tolerance to foreign particles may result in inadequate clearance of pathogens, causing an atypical and severe immune response in immunocompromised individuals.

Immunosuppressive drugs are indispensable in modern medicine, being used for the treatment of inflammatory and autoimmune diseases, as well as to suppress the rejection of transplanted organs. By their structural properties, they can be divided into small molecules that suppress the immune system by inhibiting the cell cycle or cytokine release, and biological agents (e.g., monoclonal or polyclonal antibodies) that deplete target cell populations or their function [3,4]. In addition, various environmental pollutants, such as heavy metals and pesticides, have been shown to suppress the immune system [5,6]. In this case, immunosuppression is undesirable and results in the immune system malfunction.

Traditionally, immunosuppression can be assessed using T cell-dependent antibody response assays on rodents [7], but there are tendencies to reduce the number of animal tests [8]. A three-tier approach is now proposed for the in vitro assessment of immunosuppression [2,8]. First, myelotoxicity is tested, and if the compound is myelotoxic, it is considered immunotoxic. In the second tier the lymphotoxicity is examined, and in the third tier different tests can be performed, such as assays of proliferation, T cell activation, cytokine release or testing natural killer cell activity. Although this stepwise approach is widely recognised, there is a lack of validated methods that can be used to assess immunosuppression. In 2023, a new Organisation for Economic Co-operation and Development (OECD) test guideline TG444a was published. In this guideline, luciferase signalling of IL-2 promoter is measured in 2H4-derived Jurkat cells and its decrease is attributed to immunosuppression [9,10]. Several other attempts to provide reliable in vitro models have been reported, e.g., the Multi-ImmunoTox Assay (MITA), which measures IL-2, IFNγ and IL-8-driven luciferase reporter signalling in Jurkat or THP-1-derived cells [11], the expression of the surface molecule CD86, gene marker *HMOX1* and reactive oxygen species (ROS) production in THP-1 cells [12], the activation of PBMC and consequent measurement of a wide range of markers allowing the identification of chemical effects on specific immune cell subtypes [13] and many other processes [14,15,16,17].

One of the approaches for assessing immunosuppression is the whole-blood cytokine-release assay (WBCRA), in which the whole blood is activated by lipopolysaccharides (LPS) or Staphylococcal enterotoxin B (SEB) and the released cytokines from monocytes and lymphocytes are then measured [18]. Although this method has been widely recognised and used [19,20], it suffers specific drawbacks, such as the need for ex vivo material, interindividual variability between blood donors, unstandardised monocyte/leukocyte counts, discrepancies of protocols between laboratories and reporting of results as well as unfeasibility for high-throughput applications [8,21,22].

Cell lines are commercially available and allow assays to be performed at low cost [8], but it must be considered that they are subjected to genotypic and phenotypic changes and therefore may not adequately represent the primary cells [23]. Consequently, the choice of readout marker must be carefully selected and it should resemble the in vivo situation. Typically, in vitro tests require the activation of cell lines that activate specific signalling pathways, the results of which are then measured. The use of different activators leads to the activation of different signalling pathways, and the underlying mechanisms need to be considered when interpreting the results. Furthermore, the validated cell line-based model is expected to provide robust and reliable results and avoid the intra-individual variability when using ex vivo models.

In this study, we prepared a coculture of THP-1 (monocytes) and Jurkat (lymphocytes) cell lines in order to mimic the WBCRA in vitro, allowing us to observe the sum of the immunosuppressive effects on two distinct cell types. The viability and activation of the coculture were assessed and after the application of known immunosuppressive drugs, the reduction in IL-8 secretion was selected as a biomarker of immunosuppression. Finally, a series of environmental pollutants was applied to the coculture in order to investigate their immunosuppressive potential.

## 2. Materials and Methods

### 2.1. Chemicals

Doramapimod (99.62%; CAS 285983-48-4), tacrolimus monohydrate (98.88; CAS 109581-93-3), dapsone (99.22%; CAS 80-08-0) and rapamycin (99.52%; CAS 53123-88-9) were obtained by MedChemExpress LLC, New Jersey, NJ, USA as 10 mM stocks in DMSO. Hydrocortisone (≥98%; CAS 50-23-7) was obtained from Sigma Aldrich(St. Louis, MO, USA) and prepared as 10 mM stock in DMSO (ACS grade, ≥99.9%, Sigma Aldrich). Bisphenols BPA (99.9%; CAS 80-05-7), BPE (99.9%; CAS 2081-05-5), BPAP (99.9%; CAS 1571-75-1), BPP (99.9%; CAS 2167-51-3), BPZ (99.9%; CAS 843-55-0), TCBPA (99.8%; CAS 79-95-8) and BPS-MAE (99.9%; CAS 97042-18-7) were obtained from Chiron AS, Norway and prepared as 10 mM stocks in DMSO. D-mannitol (≥98.0%; CAS 69-65-8) was obtained from MedChemExpress LLC and prepared as 10 mM stock in PBS (Sigma Aldrich, St. Louis, MO, USA).

### 2.2. Cell Lines and Cell Culture

THP-1 and Jurkat (Clone E6-1) cell lines were obtained from ATCC [24,25]. Cells were cultured in RPMI 1640 medium (Sigma Aldrich) supplemented with 10% foetal bovine serum (Gibco, ThermoFisher Scientific, Waltham, MA, USA), 2 mM L-glutamine (Sigma-Aldrich) and 1 mM sodium pyruvate (Sigma) in a humidified 5% CO_2_ atmosphere at 37 °C. For all experiments, THP-1 cells were used with a concentration of 0.6 × 10^5^ cell/mL and Jurkat cells were used with a concentration of 3 × 10^5^ cell/mL.

### 2.3. Metabolic Activity Assay

To evaluate the metabolic activity of each cell line or coculture, 100 µL of cells/well were seeded in black 96-well plates and incubated for 24 h. An amount of 10 µL of 400 µM resazurin (Sigma Aldrich) dissolved in PBS was added to the cells and further incubated for 3 h. Resorufin fluorescence was measured at λ_ex_/λ_em_ = 530/590 nm using an automated plate reader (Synergy 4 Hybrid; BioTek, Winooski, VT, USA). Two independent experiments were performed in technical duplicates.

The effect of immunosuppressive drugs on the metabolic activity of cocultures was also investigated. An amount of 100 µL of the coculture/well was seeded into black 96-well plates. On a separate plate, the bisphenols were first appropriately diluted in DMSO, then diluted in the medium and added to the cells. Concentrations between 1 nM and 50 µM were tested. As control samples, the cells were exposed to a vehicle control (0.5% DMSO). After 24 h of incubation, 10 µL of 400 µM resazurin was added and resorufin fluorescence was recorded after 3 h as described above. Three independent experiments were performed with technical duplicates.

### 2.4. Cell Stimulations and Cytokine Measurements

For the evaluation of immunosuppression, 500 µL of cells was seeded in 24-well plates. Pre-treatment with compounds or vehicle control was performed for 2 h, followed by 24 h of stimulation with 1 µM ionomycin (Sigma Aldrich), 50 nM phorbol 12-myristate 13-acetate (PMA, Sigma-Aldrich) and 10 ng/mL lipopolysaccharides from Escherichia coli O111:B4 (LPS, Sigma Aldrich). The supernatants were collected by centrifugation and stored at −80 °C until analysis. IL-8 (and screening experiments) was measured using the Human Inflammatory Cytokine Cytometric Bead Array (CBA)-I kit (BD Biosciences, San Diego CA, USA) according to the manufacturer’s instructions [26]. IL-2 and TNFα were measured by ELISA (Thermo Fisher Scientific, Waltham, MA, USA) according to the manufacturer’s instructions. At least three independent experiments were performed. Results are presented as arithmetic means with standard deviations of fold inductions compared with the vehicle control. For statistical analysis, Dunnett’s test for multiple comparisons was performed using GraphPad Prism 10.1.2. (San Diego, California, CA, USA).

## 3. Results

### 3.1. Cell Culture Viability and Activation

**Figure 1:**
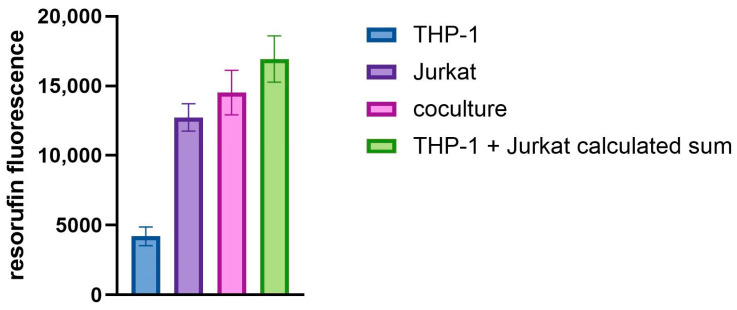
Resorufin fluorescence signals from THP-1 and Jurkat cells, their coculture and expected sum of separate cells.

The coculture was prepared from THP-1 and Jurkat cells with a ratio of 1:5, which corresponds to the ratio of monocytes to lymphocytes in the blood of healthy people [27,28,29,30,31,32,33]. With the use of a resazurin-based metabolic test, we assessed the viability of THP-1 cells, Jurkat cells and their coculture after 24 h of incubation. We assumed that a fully viable coculture would have a resorufin signal equal to the sum of the resorufin signals of THP-1 and Jurkat cells. Compared with the sum of the resorufin signals of THP-1 and Jurkat cells, the coculture produced 86 ± 1% of the calculated signal (Figure 1). We concluded that the prepared coculture was viable enough to perform further experiments.

The immunosuppressants were applied to the cocultures to assess their cytotoxicity. Based on the metabolic test, all compounds met the viability criterion of >80% at concentrations up to 10 µM (Figure S1).

Several activators were tested to activate the coculture. The Staphylococcal enterotoxins A, B and E were tested at concentrations of up to 2.5 µg/mL, but did not stimulate the secretion of the measured cytokines. The mixture of 10 ng/mL LPS, 1 µM ionomycin and 50 nM PMA as activators resulted in the release of IL-2, IL-8 and TNFα from the coculture, with THP-1 cells secreting only IL-8 and Jurkat cells secreting all three cytokines (Figures S2 and S3). LPS contributed to the IL-8 release from THP-1 cells (Figure S4).

### 3.2. Effect of Immunosuppressive Drugs on Cytokine Release

A series of five known immunosuppressants with different mechanisms of action (hydrocortisone, doramapimod, dapsone, tacrolimus and rapamycin) were selected for method validation and their effects on IL-2, IL-8 and TNFα secretion on THP-1, Jurkat cells and their coculture were evaluated. We arbitrarily set an immunosuppressive threshold at a 30% decrease in cytokine release and considered compounds that caused a reduction in cytokine release of more than 30% as immunosuppressants.

#### 3.2.1. IL-2

**Figure 2:**
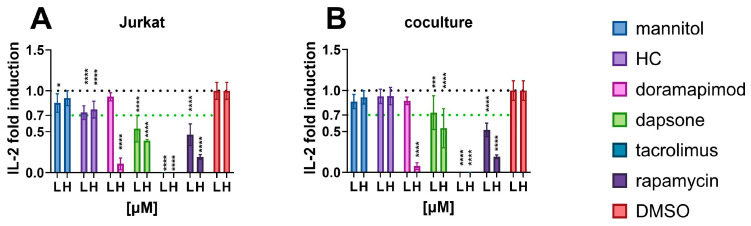
Effects of known immunosuppressants on IL-2 released from (**A**) Jurkat cells and (**B**) coculture. L—low concentration (100 nM), H—high concentration (10 µM). * *p* < 0.05; *** *p* = 0.0001; **** *p* < 0.0001.

As shown in Figure 2, all compounds decreased IL-2 secretion from Jurkat cells. Hydrocortisone (HC) was the only compound for which no dose adjustment was observed, and its modest decrease in Jurkat cells was lost in the coculture. Doramapimod only achieved statistically significant IL-2 decrease at 10 µM, while dapsone, rapamycin and tacrolimus were also effective at 100 nM. Their effects in Jurkat cells were retained in the coculture.

#### 3.2.2. TNFα

**Figure 3:**
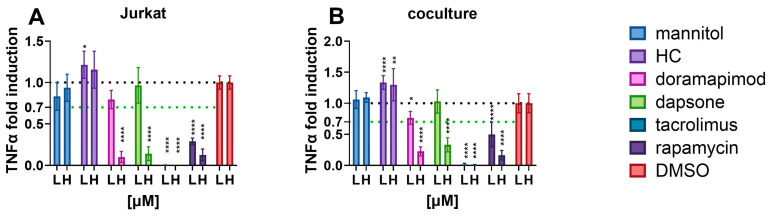
Effects of known immunosuppressants on TNFα released from (**A**) Jurkat cells and (**B**) coculture. L-low concentration (100 nM), H-high concentration (10 µM). * *p* < 0.05; ** *p* < 0.005; **** *p* < 0.0001.

Figure 3 shows the secretion of TNFα from Jurkat cells and the coculture. While doramapimod, dapsone, tacrolimus and rapamycin showed a dose-dependent inhibition of TNFα, HC significantly increased the release. Doramapimod and dapsone showed modest effects at 100 nM, but they were more effective at 10 µM concentrations. Rapamycin and tacrolimus already caused significant TNFα inhibition at 100 nM. The effects from Jurkat cells were retained in the coculture.

#### 3.2.3. IL-8

**Figure 4:**
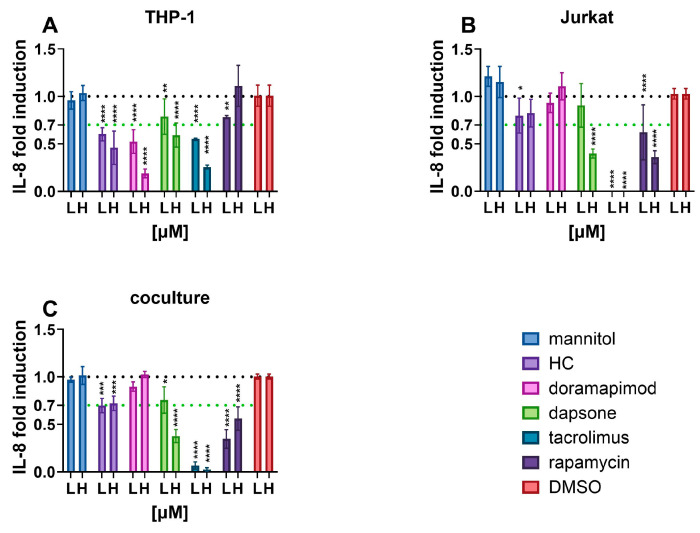
Effects of known immunosuppressants on IL-8 released from (**A**) THP-1 cells, (**B**) Jurkat cells and (**C**) coculture. L-low concentration (100 nM), H-high concentration (10 µM). * *p* < 0.05; ** *p* < 0.005; *** *p* = 0.0001; **** *p* < 0.0001.

As shown in Figure 4, THP-1 cells were easily subjected to IL-8 inhibition by all selected immunosuppressants. Interestingly, only 10 µM rapamycin failed to reduce IL-8 secretion and appeared to be a more potent inhibitor at a concentration of 100 nM. IL-8 secretion from Jurkat cells was relatively unaffected by HC and doramapimod, and dapsone was only active at 10 µM. Tacrolimus and rapamycin significantly inhibited IL-8 secretion from Jurkat cells, even at the tested concentration of 100 nM. In coculture, all immunosuppressants except for doramapimod caused a reduction in IL-8 secretion, which was dose-dependent, with the exception of HC.

### 3.3. Evaluation of Immunosuppressive Action from Environmentally Occurring Bisphenols

**Figure 5:**
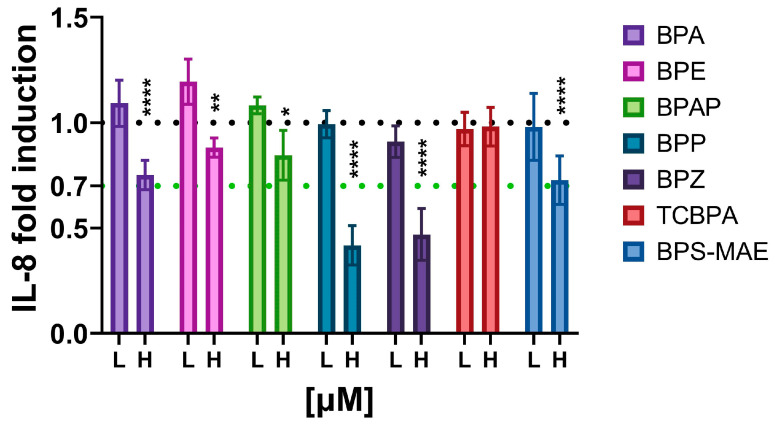
Effect of bisphenols on IL-8 secretion from the coculture. L-low concentration (100 nM), H-high concentration (10 µM). * *p* < 0.05; ** *p* < 0.005; **** *p* < 0.0001.

The coculture was exposed to bisphenol A (BPA), bisphenol E (BPE), bisphenol AP (BPAP), bisphenol P (BPP), bisphenol Z (BPZ), tectrachloro bisphenol A (TCBPA) or bisphenol S-monoallyl ether (BPS-MAE), found in a variety of plastic products, cans and thermal paper, as well as in the environment (Figure 5) [34,35,36]. At 100 nM, none of the bisphenols inhibited IL-8 secretion, while BPE even increased it significantly. At 10 µM, all compounds except BPE and TCBPA significantly decreased IL-8 secretion, where BPP and BPZ exceeded the immunosuppressive threshold of >30% inhibition.

## 4. Discussion

With the aim of reducing animal testing, regulators emphasise the importance of in silico, in chemico and in vitro approaches to reliably assess the hazards posed by chemicals [37,38]. In the field of immunotoxicology, new approach methods (NAMs) should therefore replace current assays, such as the T-cell-dependent antibody response assay on rodents and the WBCRA, which requires ex vivo material from human donors. NAMs are not necessarily newly established methods, but are implications of conventional methods that have been used and validated to assess chemical hazard [39].

In this study, we investigated whether the coculture of Jurkat and THP-1 cells can be used to reliably measure the immunosuppressive effects of chemicals. First, we investigated the viability of coculture. Although this coculture is commonly used [40,41], there is evidence of THP-1 and Jurkat incompatibility [42,43]. In our hands, the coculture prepared at a ratio equivalent to in vivo conditions exhibited more than 86% of the expected metabolic activity (Figure 1) and the cells released comparable amounts of cytokines when tested alone or in coculture (Figure S3). We therefore concluded that the coculture was viable enough to be suitable for use in further experiments.

In the WBCRA, the blood can be activated by various stimulants, such as LPS or SEB, and the measured cytokines are mainly released from lymphocytes and monocytes [18]. Our coculture was non-responsive to any Staphylococcal enterotoxin tested (Figure S2). Activation was then achieved by the combination of ionomycin, PMA and LPS. PMA is the activator of protein kinase C, while ionomycin acts as a calcium ionophore. Together, they activate the cells by bypassing the T-cell membrane receptor [44]. LPS activates the cells by binding to Toll-like receptor 4 (TLR4), which leads to the activation of various signalling pathways [45,46]. Each of the mentioned activators has been reported to be used in WBCRA [44]. In our hands, the activated coculture secreted IL-8 from THP-1 cells and IL-8, IL-2 and TNFα from Jurkat cells (Figure S3). LPS contributed importantly to the release of IL-8 from THP-1, while Jurkat cells were only activated by ionomycin and PMA (Figures S3 and S4). These cytokines have also been reported to be measured in the WBCRA [21,47,48].

To test the suitability of the coculture for the assessment of the immunosuppressive effect, we applied known immunosuppressants to the coculture and measured the released cytokines. We arbitrarily applied a >30% decrease in secreted cytokines as a positive threshold for immunosuppression. The compounds were tested at 10 µM to confirm that immunosuppression could indeed be assessed using this model, and at 100 nM to determine if effects were seen at concentrations approaching those in vivo in blood (therapeutical concentrations for HC up to 70 nM [49], dapsone up to 68 nM [50], tacrolimus up to 54 nM [10] and rapamycin up to 16 nM [10,51]). The immunosuppressants used in this study have different mechanisms of action—HC is a glucocorticoid steroid, doramapimod is a p38 MAPK inhibitor, dapsone is a myeloperoxidase (MPO) inhibitor, tacrolimus is an inhibitor of calcineurin phosphatase and rapamycin is an mTOR inhibitor.

In the presence of tacrolimus, ionomycin-induced intracellular Ca^2+^ cannot bind to calcineurin and consequently activate NFAT, which regulates IL-2 [52], IL-8 [53] and TNFα [54]. Not surprisingly, all cytokines were completely inhibited in Jurkat cells, while some IL-8 was still released from THP-1 cells, indicating the different underlying activation mechanisms in the cell types. While Jurkat cells are primarily activated by ionomycin and PMA, LPS acts on multiple signalling pathways in THP-1, including NFκB, AP-1 and MAPK, all of which can regulate IL-8 [55,56,57].

p38 MAPK can be induced by LPS [55] or ionomycin and PMA [58] and its downstream pathways include the activation of transcription factors such as NFκB, AP-1 and NFAT that regulate the transcription of various cytokines. Doramapimod inhibited IL-2 and TNFα by more than 30% at 10 µM, and its modest effects were also observed at lower concentrations. Interestingly, even at 100 nM, it reduced IL-8 from THP-1 monocytes below the threshold of immunosuppression, while it showed no effect on IL-8 released from Jurkat cells and coculture. The decrease in IL-2 and TNFα from Jurkat cells supports its binding to the target p38 MAPK, and the decrease in IL-8 from THP-1 cells confirms the involvement of p38 MAPK in IL-8 regulation. The nature of insensitive IL-8 in Jurkat cells and coculture remains speculative and requires further investigation.

Inhibitors of mTOR, such as rapamycin, are used to block cellular proliferation through the inhibition of activation proteins, such as kinase S6 [59]. In THP-1 cells, IL-8 was only slightly reduced by rapamycin, which is less than expected compared with previously published results [60]. On the other hand, the inhibition of the PI3K/Akt/mTOR signalling pathway by rapamycin was observed in Jurkat cells and in the coculture, where rapamycin significantly reduced IL-2, TNFα and IL-8 below the limit of immunosuppression at both concentrations tested.

Dapsone is an MPO inhibitor that downregulates the intracellular ROS of neutrophils and thus inhibits their action. It has also been shown to reduce the release of proinflammatory cytokines, such as IL-8 from keratinocytes and bronchial epithelial cells [61,62], as well as TNFα in various models, where it acts by downregulating TLR4 and dephosphorylating NFκB [63,64]. In our hands, it affected IL-2, TNFα and IL-8 in all cell models and was able to exceed the immunosuppression threshold at 10 µM.

HC is an interesting compound that slightly inhibited IL-2 secretion from Jurkat cells without dose dependence, but it was completely inactive in coculture. Kimura et al. [10] observed that the glucocorticoid dexamethasone downregulated IL-2 expression in their in vitro model, but was unable to pass the immunosuppression threshold at doses below therapeutical concentrations (0.088 µg/mL). In T cells, IL-2 expression is subjected to regulation by the glucocorticoid receptor and AP-1 [65], but Jurkat cells have been reported to have a mutation in the GR and we speculate that this mutation might be the reason for Jurkat insensitivity [66,67]. A similar behaviour in Jurkat cells was also observed for IL-8, while a greater decrease was observed in THP-1 cells and likely consequently in the coculture. Interestingly, TNFα was increased by HC. The underlying mechanisms of glucocorticoid-mediated proinflammatory response could be attributed to the increase in nitric oxide [68] or enhanced ATP-dependent secretion of TNFα [69].

Understanding the mechanisms leading to changes in cytokine secretion in each cell line is critical for assessing whether the cell model is suitable for measurements of immunosuppression. We have shown that both cell lines respond to a variety of immunosuppressants, with THP-1 cells responding moderately to mTOR inhibitors and Jurkats to glucocorticoids and p38 MAPK inhibitors. As IL-8 is the only cytokine released by both cell types in coculture, we selected IL-8 as a marker of immunosuppression for our model and set a positive threshold of 30% of its inhibition. IL-8 is a chemotactic agent that is mainly released by monocytes, macrophages, epithelial cells and also CD4+ lymphocytes. It attracts neutrophils and T cells to the site of infection [70,71], induces exocytosis of granules and histamine as well as a respiratory burst in the target cells [72]. Its overexpression is associated with inflammatory diseases and IL-8 neutralising antibodies are being investigated for their treatment [70,73]. In in vitro tests, the IL-8 Luc assay in combination with MITA [22] has shown promise as a tool for detecting immunosuppression, as has the measurement of IL-8 in WBCRA [18,47,48]. Therefore, its reduced secretion is a suitable marker for the detection of immunosuppression.

The established coculture model is able to recognise a range of immunosuppressants (HC, dapsone, tacrolimus and rapamycin) with different mechanisms of action, while it is not able to recognise p38 MAPK inhibitors. Whether it can also recognise other inhibitors of the MAPK family, such as ERK and JNK inhibitors, will be further investigated in our laboratory. It must be noted that the arbitrarily set threshold for the positive detection of immunosuppression was generally reached in the micromolar range, exceeding therapeutical concentrations by 100 to 1000 times. Nevertheless, it avoids the need for in vivo and ex vivo testing, and is standardised, robust and easy to use in laboratories.

As evidence accumulates that BPA has toxic effects, its use is increasingly restricted by law [74,75]. Recently, due to the fact that BPA affects Th17 cells in rodents, which can lead to inappropriate immune stimulation, the EFSA has reduced its tolerable daily intake from 4 µg/kg to 0.2 ng/kg body weight in 2023 [76]. To overcome the legal obstacles, several BPA analogues are now appearing in consumer products, but without a full toxicological assessment of whether they are actually safer than BPA [77,78,79]. In addition to the well-known endocrine effects of bisphenols, several studies to date have shown that they also modulate the immune system [80] and their effects are likely cell type dependent. Macrophages are primarily exposed to the proinflammatory effects of bisphenols (e.g., BPA, bisphenol S and bisphenol F) as they promote M1 polarisation, while their effect on proinflammatory cytokines release depends on the concentration tested [80,81]—for example, BPA was found to upregulate several cytokines at 100 nM and 10 µM, but to downregulate them at 1 µM and 100 µM [81]. On the other hand, BPA and bisphenol S suppress IL-8 secretion by neutrophil granulocytes (at 1 nM) and THP-1 monocytes (at 10 µM), thereby exerting immunosuppressive potential [82,83], while IL-2 secretion from Jurkat cells is not affected by BPA (in nano- and micromolar concentrations) [84].

Therefore, we applied a set of seven bisphenols to our coculture model to observe their effects on IL-8 release. In 2021, the European Chemicals Agency (ECHA) added BPE, BPAP, BPP, BPZ and BPS-MAE to the list of compounds, for which hazard cannot be clarified due to the lack of available data [78]. Indeed, data on how these chemicals affect the immune system are scarce, but the existing evidence from studies on mice suggests that BPA analogues promote inflammatory responses. Exposure to BPP leads to inflammation in the intestines with increased expression of proinflammatory IL-1β, IL-6 and TNFα [85]. Early life exposure to BPAP was found to increase the proportion of macrophages and activation of dendritic cells [86]. On the other hand, in TCBPA-exposed mice, the decrease ratio of CD3+ T cells to regulatory T cells was observed, indicating the immunosuppressive effects. However, the serum levels of both pro- and anti-inflammatory cytokines were elevated [87].

As shown in Figure 5, none of the bisphenols tested in our model had an immunosuppressive effect at 100 nM, while BPE even increased IL-8 at this concentration. BPA significantly decreased IL-8 at 10 µM, although the mean of the replicates did not exceed 30% inhibition. This finding aligns with existing data about BPA’s effect on THP-1 and neutrophil granulocytes [82,83]. BPS-MAE showed comparable immunosuppressive potential to BPA, while BPP and BPZ were more potent and reduced IL-8 secretion by more than 30% when tested at 10 µM. Their efficacy at this concentration was comparable to that of dapsone, while they were more effective than HC or rapamycin (4). On the other hand, BPAP, BPE and TCBPA did not show immunosuppressive mechanisms in our model.

The endocrine effects of bisphenols in in vitro studies were observed in the nano- to low micromolar range. Specifically, the EC_50_ values for estrogenic activities are 0.86 µM for BPA [88] and 0.124 µM for BPZ [89], while antiestrogenic potency of BPP is observed at IC_50_ = 1.9 µM [89]. The fact that, in our in vitro model, immunosuppressive effects were observed at 10 µM (but not at 100 nM), which exceeds the determined estrogenic parameters, suggests that immunosuppression might be a consequence of endocrine disruption, as well as other intracellular effects.

In comparison with previously published studies, our observations that BPP has a strong anti-inflammatory effect and that BPAP and TCBPA do not modulate the release of IL-8 from the coculture highlight the pleiotropic nature of bisphenols and their cell type-dependent mechanisms, as well as the importance of a judiciously chosen in vitro model for the determination of immunomodulation by these compounds. Based on our observations that BPP and BPZ are even more effective than BPA in reducing IL-8 release; we strongly recommend using these substitutes with caution.

## 5. Conclusions

In this study, we investigated the suitability of the coculture model of THP-1 and Jurkat cells as a NAM for the in vitro assessment of immunosuppressive mechanisms caused by chemicals. After verifying the viability of the coculture and optimising its activation, we exposed it to a set of immunosuppressive drugs and investigated their effects on cytokine release. We selected IL-8 as a marker for immunosuppression and with this setup, we were able to detect immunosuppressants with different mechanisms of action, except for p38 MAPK inhibitors. Furthermore, by applying a set of environmentally occurring bisphenols to the model, we showed that BPS-MAE had comparable immunosuppressive potential to BPA, while BPP and BPZ were even more potent. Our model avoids the need for ex vivo materials, is standardised in execution and is easily applicable in other laboratories. To increase the usefulness of this model, it is worth exploring further whether it is able to recognise a broader range of immunosuppressants with different mechanisms of action and make it suitable for high-throughput applications.

## Data Availability

Data is contained within the article or supplementary material.

## Supplementary Materials

The following supporting information can be downloaded at: https://www.mdpi.com/article/10.3390/toxics12080607/s1, Figure S1: Viability of the coculture upon application of immunosuppressive drugs for 24 h.; Figure S2: Release of cytokines from the coculture after 24 h of stimulation with different activators; Figure S3: Release of cytokines from THP-1 cells, Jurkat cells and coculture; Figure S4: Effect of LPS on the release of IL-8.
