# Supplementary material for: Evaluation of THP-1 and Jurkat Cell Lines Coculture for the In Vitro Assessment of the Effects of Immunosuppressive Substances
Source: Toxics. 2024 Aug 19;12(8):607. doi: 10.3390/toxics12080607 (PMC11358983; doi:10.3390/toxics12080607)
Supplement: Supplementary file 1 [file toxics-12-00607-s001.zip › toxics-3128176-supplementary.pdf]

Evaluation of THP-1 and Jurkat cell lines coculture for the in vitro assessment of the effects of immunosuppressive substances

Nina Franko, Marija Sollner Dolenc

SUPPLEMENTARY MATERIAL

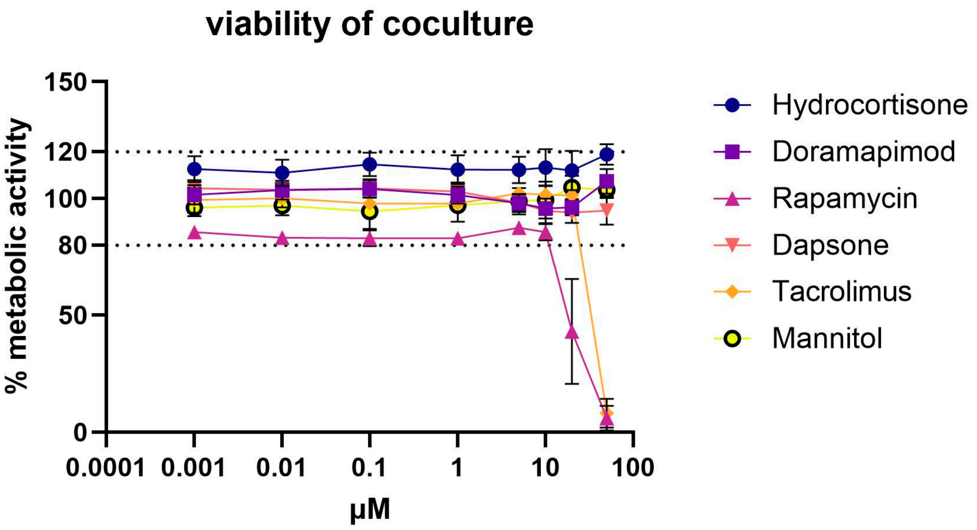

Figure S1: Viability of the coculture upon application of immunosuppressive drugs for 24 h.

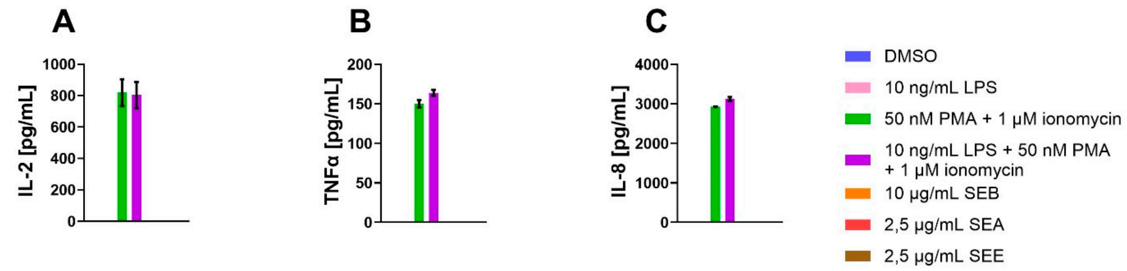

Figure S2: Release of cytokines from the coculture after 24 h stimulation with different activators. Panel A) release of IL-2; Panel B) release of TNFα; Panel C) release of IL-8.

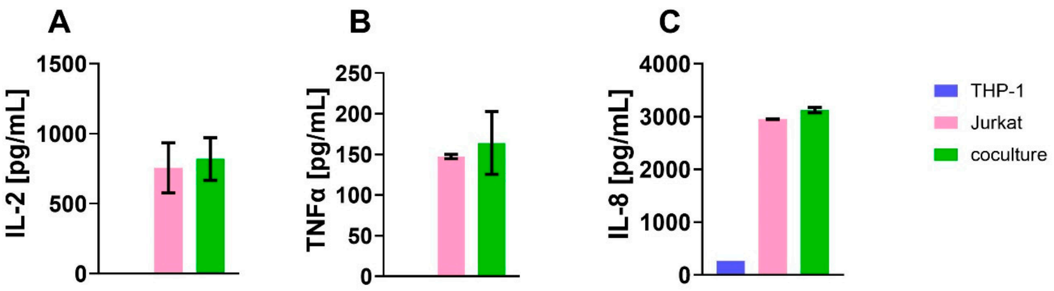

Figure S3: Release of cytokines from THP-1 cells, Jurkat cells and coculture, activated with 1 μM ionomycin, 50 nM PMA and 10 ng/mL LPS. Panel A) release of IL-2; Panel B) release of TNFα; Panel C) release of IL-8.

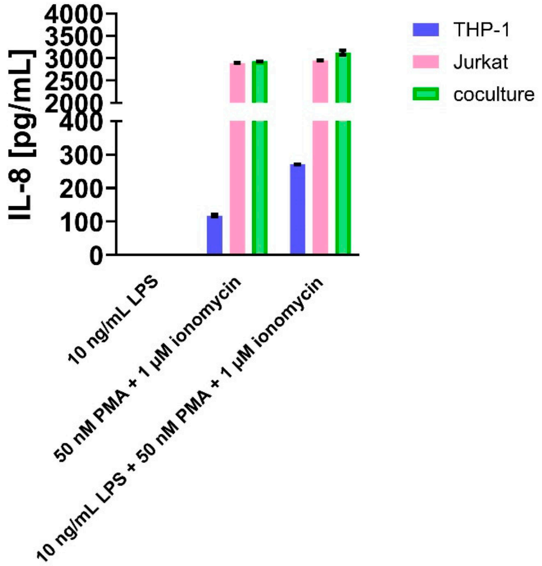

Figure S4: Effect of LPS on the release of IL-8.
